# Community Pharmacist’s Role in Detecting Low Back Pain, and Patient Attitudes—A Cross-Sectional Observational Study in Italian Community Pharmacies

**DOI:** 10.3390/ijerph17165965

**Published:** 2020-08-17

**Authors:** Corrado Giua, Paola Minghetti, Giorgio Gandolini, Paolo Rocco, Elisa Arancio, Teresa Bevacqua, Nicolina Floris, Enrico Keber, SGCP, Umberto M. Musazzi

**Affiliations:** 1 Società Italiana Farmacia Clinica (SIFAC), Viale Regina Margherita 30, 09126 Cagliari, Italy; corrado.giua@gmail.com (C.G.); elisaa8750@hotmail.it (E.A.); terbevacqua@gmail.com (T.B.); ni.26@hotmail.it (N.F.); enrico.keber@sifac.it (E.K.); segreteria@sifac.it (SGCP); 2 Department of Pharmaceutical Sciences, Università degli Studi di Milano, Via G. Colombo 71, 20133 Milan, Italy; paola.minghetti@unimi.it (P.M.); paolo.rocco@unimi.it (P.R.); 3 Centro Reumatologia, IRCCS Santa Maria Nascente, Fondazione Don Gnocchi ONLUS, 20148 Milano, Italy; giorgio_gandolini@yahoo.it

**Keywords:** low back pain, community pharmacy, Roland and Morris disability questionnaire, start back screening tool

## Abstract

Background: Low back pain (LBP) is one of the most frequent diseases for which patients seek advice in a community pharmacy. The study aimed to evaluate the feasibility of the administration by community pharmacists of questionnaires to assess the LBP intensity and disability degree in patients entering community pharmacies and the attitudes they have toward pain management by pharmacological and non-pharmacological strategies. Methods: An explorative, cross-sectional, observational, and quantitative study was performed. Twelve Italian community pharmacists were asked to submit a questionnaire on LBP to patients visiting their pharmacies. The questionnaire included a pain intensity scale, and two validated tools: the Roland and Morris Disability Questionnaire (RMDQ) and the Start Back Screening Tool (SBST) to determine the degree and risk of patient disability, respectively. Results: 872 patients filled out the questionnaires in 6 months. No statistical differences between genders (*p* > 0.30) were recorded for pain intensity (Female: median score 6, IQR 4–7; Male: median scores 5, IQR 4–7; *p* > 0.30) and disability associated with LBP (RMDQ high-disability level: Females, 14.7%, Males, 15.0%; *p* > 0.90). Most of the patients (69%) reported a low degree of disability, but the risk of disability was medium and high in 36% and 18% of them, respectively (*p* < 0.05). About 14% of patients declare to never seek for physician’s advice despite their medium-high degree of disability. Conclusion: The study demonstrated the feasibility of validated tools for assessing the degree and risk of disability in LBP patients administrable in community pharmacies. Moreover, the community pharmacy resulted in an important care portal for patients suffering from moderate LBP and for intercepting patients who suffered from severe LBP but had never reported their problem to their physician.

## 1. Introduction

Low Back Pain (LBP) is a disease of the dorso-lumbar region of the back, between the inferior margin on the scapula and the buttocks. LBP is one of the biggest public health problems in Western countries [1] and one of the leading causes of reduction in patients’ quality of life worldwide [2]. In industrialized countries, 67% and 84% of citizens have been affected by LBP at least once in their lifetime [3,4]. The prevalence rate increases from childhood to adolescence peaking between 35 and 55 years of age [5]. If LBP is not properly treated, the symptoms generally worsen, and risks of clinical complications increase. Indeed, patients affected by LBP often report functioning issues and difficulties in participating in daily activities, with impairments in interpersonal relations [6]. Moreover, LBP-induced disability has important consequences on healthcare expenditures and the working capacity of patients [7]. 

Due to its high prevalence, LBP represents one of the most frequent causes (the fifth cause in the USA) of patients’ visits to the General Practitioner (GP) [8] and one of the most frequent clinical problems reported by the patients to community pharmacists (CPs). In this context, CPs can counsel patients on the most appropriate self-medication strategy or support them in following the medical prescription correctly. Indeed, in Italy, like in other countries, non-prescription medicines are generally products authorized for the treatment of passing or minor disturbances that can be bought autonomously by patients or counseled by the CPs. On the contrary, prescription medicines are dispensed by the CPs only following a medical prescription written by a GP or a specialist based on a medical diagnosis.

However, the role of the CP does not need to be limited to dispensing. Indeed, the CP represents an under-exploited resource, albeit potentially very useful and proactive in the assessment, in the first instance, and management of LBP. Their widespread presence in any geographical area and frequent contact with patients potentially provide the CPs with many support tools, such as the possibility of establishing educational support interviews, performing follow-ups, and monitoring the progress of implemented interventions [9,10,11,12,13]. Defining validated modes and tools to help support the patient with LBP is critical. To date, there are few available data collected in pharmacies in which patients with LBP seek the advice of CPs to manage their disease.

The study aims to determine the impact of LBP on disability degree and risk of future disability in patients entering a community pharmacy, record to whom patients trusted for advice on the pharmacological treatments taken to treat LBP and review the strategies used in managing different LBP of different severity. Moreover, a secondary objective was to investigate the feasibility of administration of validated questionnaires to patients who suffer by LBP and rely on self-medication by Italian CPs. 

## 2. Materials and Methods 

An explorative, cross-sectional, observational, and quantitative study was carried out. The study was conducted within the first semester of 2016 by 12 CPs collectively named “The SIFAC Group of clinical Community Pharmacists” (SGCP), in 12 community pharmacies distributed homogeneously across Italy. Preliminarily, all CPs participated in an educational workshop divided into two sections. The first (4 h) was held by a rheumatologist and gave CPs a specific and detailed training on LBP symptomatology and its evidence-based management [13]; the second (1.5 h) was focused on the study protocol, patient recruitment, and data collection.

Data were gathered exclusively using a paper questionnaire of 58 questions, subdivided into five sections: (1) Patient socio-demographic features and behavioral habits, (2) Pharmacological treatments and clinical history of the LBP; (3) estimation of pain intensity by the Numeric Pain Rating Scale (NPRS) [14,15]; (4) Start Back Screening Tool (SBST) [16]; (5) Roland and Morris Disability Questionnaire (RMDQ) [17,18]. 

In Section 2 of the questionnaire, each patient was asked to report if he/she had taken oral or topical medicines for treating the LBP before the interview. For each subgroup of treatment, patients had also to report by whom the medicine had been prescribed/suggested, namely: physicians, pharmacists, others (including himself/herself). Further information (e.g., type of used active pharmaceutical ingredient, regimen) was not investigated in the study. No distinction between non-prescription and prescription medicines was made in the questionnaire. However, it is understood that the physicians may have prescribed both prescription and non-prescription medicines to patients, whereas those suggested by CPs or by others are all over the counter (OTC) medicinal products. 

In Section 3 of the questionnaire, the estimation of pain intensity was carried out using a Numeric Pain Rating Scale (NPRS) ranging from 0 (pain absence) to 10 (the worst pain possible). The patients were asked to report the pain intensity at the interview, and the minimum and maximum intensity experienced within 24 h before the interview.

Both SBST (Section 4) and RMDQ (Section 5) have been validated in their Italian versions [19,20]. The SBST was used as a prognostic tool for determining the risk of future disability of the patient [16]. The SBST provides information about the risk of disability of a specific patient based on the extent of the pain experienced in the two weeks before the completion of a self-compilation questionnaire. It categorizes patients with LBP into three levels depending on the score obtained: low, medium, and high risk of persistent disability. Indeed, the SBST is composed of 9 questions about referred leg pain, comorbid pain, disability (2 items), irritation, pain catastrophizing, fear, anxiety, and depression. The latter 5 questions are identified as a psychosocial subscale. Each question scores 1. Patients scoring 4–5 on the psychosocial subscale were classified as “high disability risk”. For scores lower than 4 on the psychosocial subscale, the patients were classified as “medium disability risk” for overall scores ≥4, and as “low disability risk” for overall score range 0–3. The RMDQ, which is one of the most used validated tools for assessing LBP, was used to scale the degree of disability generated by LBP in daily life [21]. The RMDQ is composed of 24 questions, each with score 1. The overall sum represents the final score of the questionnaire that could range from 0 (no disability) to 24 (severe disability): up to 9 the patient is classified as “with a low disability”, between 10 to 13 as “with a medium disability”, and over 14 as “with a high disability”.

The questionnaire of 58 questions was administered to patients by the CPs adhering to SGCP. Each SGCP pharmacist recruited all eligible patients entering their pharmacy. A number of 25 patients was set as a recruitment minimum goal for each community pharmacy. Eligible patients were people suffering from LBP who asked for an over-the-counter drug to mitigate it, regardless if they were regular customers of the pharmacy or not. Exclusion criteria for patient recruitment were an age lower than 18 or higher than 65 years old, pregnancy, and severe diseases of the spinal column (e.g., fractures, infective diseases, cancers). The presence of other concurrent acute and chronic diseases was not considered an exclusion criterion of the study. After obtaining the informed consent for the study, the CPs asked the patients to fill out the questionnaires. After completion, the information was entered by the CPs into a standardized web-based platform and recorded in a Microsoft® Excel 2007 file (Microsoft, Redmont, Washington, USA). 

Mean and standard deviation (SD) were used as descriptive statistics for continuous variables, while median and interquartile range (IQR) were used for reporting ordinal data and absolute and relative frequencies for nominal data. The relationships between continuous and categorical variables were evaluated using Student’s T-Test and Pearson’s chi-squared test, respectively. The results on pain intensity, RMDQ, and SBST levels were stratified based on gender, age, and BMI. The 33% and 66% percentile of the total patients’ distribution were used to determine the patient’s age levels. The data were fully managed and analyzed by using JMP^®^ 14 (SAS Institute, Cary, NC, USA). 

The study protocol was approved by the Scientific Committee for verification and control of the association’s activities of the Italian Society of Clinical Pharmacy (Società Italiana Farmacia Clinica, SIFAC; Ethical Approval No. 04G2020).

## 3. Results

A total of 894 patients who entered the 12 community pharmacies were asked to participate in the study. A percentage of 97.5% of them (*n* = 872) were recruited, whereas 22 patients did not give their consent to the study or were not eligible based on the exclusion criteria. The characteristics of the sample population are summarized in Table 1. The median age of patients was 46 years (IQR: 20). Body mass index (BMI = weight/height^2^) was stratified according to the NIH criteria [22] and revealed that 470 patients (53.9%) were normal weight, 302 (34.6%) were overweight and 100 (11.5 %) were obese. Most subjects (*n* = 660, 75.7%) were nonsmokers and declared not to be habitual alcohol consumers (80.7%), namely who take alcohol frequently or several times a week with meals. The prevalence of habitual alcohol consumers was higher among males than females (*p* < 0.0001).

No difference was reported between males and females in mean LBP duration and event incidence in the 6 months before the interview (Table 2). Patients reported that the LBP causing their visit to the pharmacy had a median duration of one week. This shows a high prevalence of acute LBP symptomatology in patients visiting community pharmacies.

Among the 872 patients who filled out the questionnaire, 70% of them had experienced another LBP event in the 6 months preceding the pharmacy visit. The percentage slightly decreased to 67.7% for patients that experienced an acute LBP (duration less than 7 days), showing that most of the recruited patients cohabited with repeated LBP events in their daily life. Moreover, it is noteworthy that the interview evidenced about 20% of patients with chronic LBP (duration higher than 12 weeks) [23]. Indeed, 11.1% of patients reported that they had been affected by LBP for more than 12 weeks, while 6.4% of them for more than a year. In this context, significant differences were observed based on patients’ gender: females experienced chronic LBP (i.e., duration higher than 12 weeks) more frequently than males (12.2% vs. 9.7%).

### 3.1. Pain Intensity, and Disability Level

**Figure 1:**
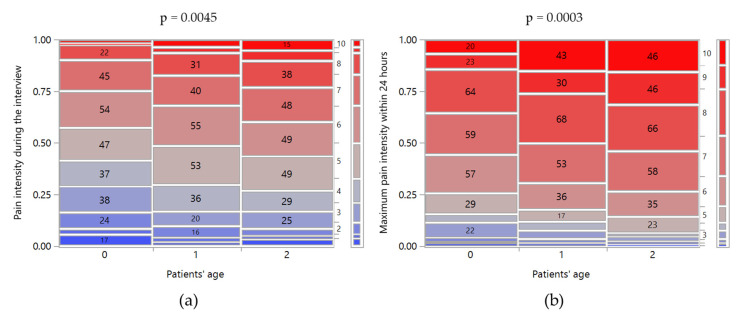
Mosaic plots of scores of (**a**) pain intensities during the interview and (**b**) maximum one within 24 h before versus patient’s age levels: 0 < 40 years old; 1, 40–52 years old; 2, >52 years old. The class counts were reported inside the mosaic plots. Above the graph is the *p*-value of Pearson’s chi-squared test between the two variables.

As shown in Table 2, most of the patients experienced a moderate-intensity LBP (score IQR: 4–7) at the interview, with small pain fluctuations within the 24 h before. No significant differences were reported between genders or between patients suffering from acute and chronic LBP. On the contrary, stratification by patient’s age suggested that pain intensity increased with age (Figure 1). For example, the percentage of patients that reported pain intensity higher than 7 during the interview was 9.7% among patients younger than 40, increasing to 17.5% and 23.2% for patients aged between 40 and 52 and more than 52 years old, respectively. A similar trend was observed for both the minimum and maximum pain intensity reported within 24 h before the interview.

**Figure 2:**
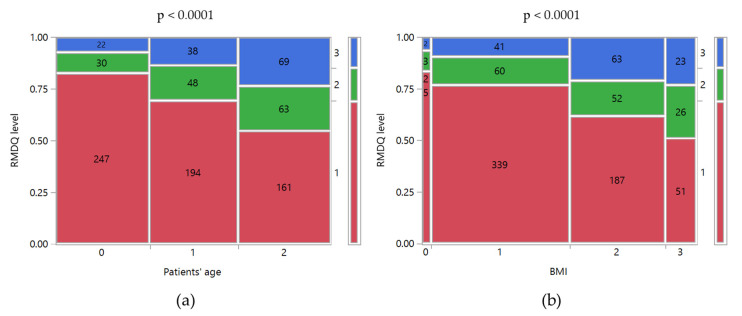
Mosaic plots of RMDQ level versus (**a**) Patient’s age and (**b**) BMI. RMDQ levels: 1, low; 2, medium; 3, high. Patients’ age levels: 0, <40 years old; 1, 40–52 years old; 2, >52 years old. BMI levels: 0, underweight; 1, normal weight; 2, overweight; 3, obese. The class counts were reported inside the mosaic plots. Above the graph is the *p*-value of Pearson’s chi-squared test between the two variables.

The application of RMDQ allowed us to determine the patient’s disability at the time of its compilation. Only 14.8% of the patients could be classified as having a high disability degree, requiring an experienced medical specialist for the management and treatment of the disease. No significant differences were observed based on gender (*p* = 0.9543), whereas significant relationships were found with the patients’ age and BMI (*p* < 0.0001; Figure 2). In particular, 7.4% of the patients younger than 40 were classified as “with a high disability”, whereas the percentage was more than double for the older ones (Age 40–52 years old: 13.6%; Age > 52 years old: 23.55%). Moreover, a higher BMI was associated with a higher disability level. Indeed, 77% of normal-weighted patients reported a low-disability level, and only 9.3% a high-disability level. For obese patients, the low and high disability levels were 51% and 23%, respectively. Finally, it is noteworthy that 10.1% and 4.1% of patients with a medium and high degree of disability, respectively, had never consulted a physician about their clinical condition.

**Figure 3:**
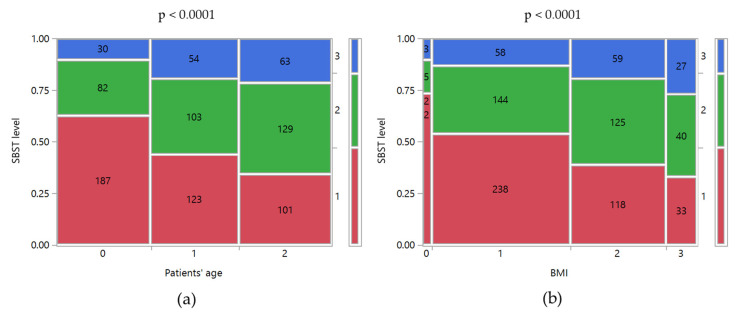
Mosaic plots of SBST levels versus (**a**) Patient’s age and (**b**) BMI. SBST levels: 1, low; 2, medium; 3, high. Patients’ age levels: 0, <40 years old; 1, 40–52 years old; 2, >52 years old. BMI levels: 0, underweight; 1, normal weight; 2, overweight; 3, obese. The class counts were reported inside the mosaic plots. Above the graph is the *p*-value of Pearson’s chi-squared test between the two variables.

The data collected through the SBST suggested that a significant percentage of patients had a medium-high risk of developing disability in the future. As shown in Table 2, 36.0% of patients are classified as medium risk and 16.9% as a high risk. As observed for RMDQ, both age and BMI increased the risk of disability (Figure 3). For example, moving from normal weight to obese patients the percentage of them with a high risk of disability increased from 13.2% to 27.0% (*p* < 0.0001). A similar trend of the high risk of disability was observed by age: 10% in patients younger than 40 years old, 19.3% in patients of 40–52 years old, and 21.5% in those older than 52 years old (*p* < 0.0001). Moreover, it is noteworthy that 5.1% of patients with a high risk of disability had never consulted a physician about their clinical condition.

The analysis of the SBST data stratified by those obtained by RMDQ permitted us to identify the patients with a significant risk of deterioration of the clinical conditions according to their disability degree. Indeed, among patients in the RMDQ level “low disability”, 29.4% showed a medium risk of future disability, whereas the risk was high for 7.1%. Additionally, 29.7% of patients with a medium disability could experience a deterioration of their clinical conditions based on the SBST level.

### 3.2. Attitudes of Patients toward LBP Management and Treatment

As shown in Table 1, patients reported different attitudes toward the management of LBP. Many of them (63.8%) had consulted a physician (GP or a specialist in orthopedics, rheumatology, traumatology) about their clinical issue. Patients older than 40 years old reported a higher propensity to consult the physician than younger ones (70% vs. 50%; *p* < 0.0001). Additionally, the physician was consulted by 86.6% of patients with chronic LBP and by 60.9% of patients with LBP with a duration lower than 12 weeks (*p* < 0.0001). On the contrary, 13.4% of patients with chronic LBP had never consulted a physician. Among other approaches, pharmacological treatments were the ones most frequently used. As shown in Table 1, oral and topical medicinal products were taken by about 70% and 50% of the patients for managing their LBP. The percentage of medicines’ users was higher in patients that consulted a physician. Indeed, 74.26% and 67.5% of who took oral and topical medicines had also consulted their physician, respectively. As expected, the majority of medicines taken by patients who consulted their physician were prescribed by the physician himself (80.6% of oral medicines; 65.5% of topical products), whereas the CPs counseled oral and topical products in 8.4% and 33.1%, respectively. On the contrary, the percentage of oral and topical medicines counseled by the CPs reached 37.3% and 45.4% of patients who took medicines without a medical diagnosis. 

**Figure 4:**
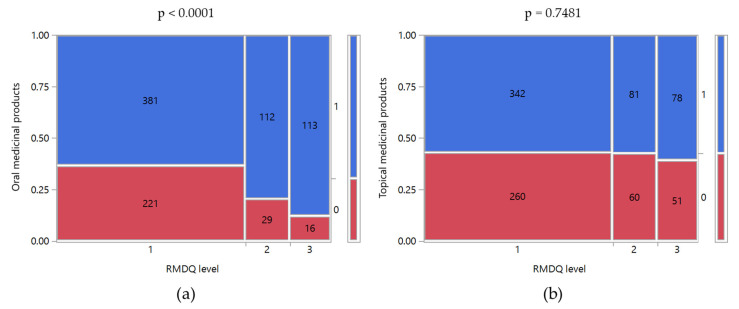
Mosaic plots of patients’ taking of (**a**) oral and (**b**) topical medicinal products to manage LBP versus RMDQ level. Taking of Oral/Topical medicinal products: 0, no; 1, yes. RMDQ levels: 1, low; 2, medium; 3, high. The class counts were reported inside the mosaic plots. Above the graph is the p-value of Pearson’s chi-squared test between the two variables.

The higher the reported pain, the higher the number of patients who consumed oral medicines to manage it (*p* < 0.0001). Interestingly, no differences were observed for topical medicinal products (*p* = 0.2046). Similar trends were observed for RMDQ levels (Figure 4). Patients taking oral medicines among those with a low-disability degree were 63.2% and reached 87.6% for a high disability degree. On the contrary, the topical products were taken by 60–70% of the patients, regardless of their disability. However, the higher the degree of disability, the higher the percentage of medicines taken by the patients based on medical prescription or counseling from CPs (*p* < 0.0001). For example, 44.8% and 22.7% of patients with a low degree of disability have taken topical medicines prescribed by a physician and counseled by CPs since the interview, respectively. Such percentages reached 23.7% and 37.5% for patients with a high degree of disability. A similar trend was observable for oral medicines, even if the percentage shift was even more significant. The percentage of oral medicines prescribed by physicians increased from 55.3% to 79.5% for patients with low and high disability degree, whereas the medicines counseled by pharmacists reduced from 21.3% to 3.6%. 

Focusing on non-pharmaceutical strategies to manage the LBP symptomatology, 39.3% of the patients carried out physical exercises and 32.7% visited a physiotherapist. The percentage of patients visiting both a physician and physiotherapist is slightly higher in women than men (31.4% vs. 23.4%, Table 1). Attitude to carry out physical exercises was higher in patients who had also visited a physiotherapist (64.9%) in comparison to patients who had visited only a physician/specialist (42.8%). Moreover, it is higher in those affected by chronic LBP (56.7%) than others (37.2%; *p* = 0.0002). A similar trend was observable for the attitude to visit a physiotherapist: patients that experienced LBP for longer periods were more inclined to resort to the physiotherapist (acute LBP, 29.2% vs. chronic LBP, 60.8%; *p* < 0.0001) as well as who had a high degree of disability (low disability: 29.4%; high disability: 43.4%; *p* = 0.0045). 

Interestingly, the results showed that the patients’ attitude to visit the physiotherapist was also driven by patients’ gender (Table 1). The females (36.5%) were more frequent users than males (28.1%; *p* = 0.0107).

## 4. Discussion

The overall results highlighted that patients visiting the pharmacy show a high prevalence of acute LBP symptomatology, with moderate intensity. The data showed that most of the patients (69%) experienced a low disability level related to their LBP. Unlike patients with high disability levels, which required experienced specialists for managing properly their pain, patients with low and medium intensity LBP were commonly taken into care by the GPs or the CPs. In this context, community pharmacies provide a unique place to collect and interpret health status information of patients. Indeed, over the last few decades, there has been an increasing interest in the CPs’ involvement in non-dispensing roles [10,11,24]. Focusing the attention on musculoskeletal disorders, LBP has been the most frequent disease reported by patients to CPs (55%) [25] and CPs have been in the ideal position to improve the quality of care of patients. On the one side, their role has been essential in medicine review use programs [24,25], monitoring drug therapies prescribed by physicians and limiting drug-related problems, such as inappropriate drug therapies, adverse drug reactions, inappropriate compliance. On the other hand, they could provide advice on the most appropriate OTC pharmacological treatments or information to guide the patients toward more appropriate and healthy behavior. Moreover, they could triage the clinical symptomatology of patients, and provide information and advice with patients with “red flags” to seek medical care. 

In comparison to other studies on LBP that focused on the patients’/CPs’ attitudes and knowledge toward LBP and its treatments [26], this article focused on patients’ assessment based on the LBP intensity and estimating the risk of disability. The results showed that CPs were able to implement validated tools for LBP assessment in their daily activities. Pain intensity scales and RMDQ were effective in supporting the CPs in the assessment of the patients’ clinical condition and rationalize their advice on the most OTC treatments. Indeed, as expected, the study underlined that both pain intensity and disability degree affected the use of analgesic medicines by patients. Interestingly, such factors have a different impact based on the type of considered dosage forms. The higher the pain intensity or disability degree of the patient, the higher the number of patients who use oral analgesics to manage their pain. On the contrary, topically applied dosage forms (e.g., foams, creams, medicated plasters) containing NSAIDs (e.g., diclofenac) were used by half of the patients regardless of their clinical conditions. The resulting stratification by those who prescribe/counsel pharmacological treatments underlined that topical treatments were more frequently counseled by CPs than oral treatments to patients, regardless of whether they have visited (33.1%) their physician or not (45.4%). However, the percentage of both oral and topical medicines taken on the CPs counsel decrease in favor of the physician prescription with the increase of the severity of the patient clinical. This might suggest a higher percentage of prescription medicines used by the patients than OTC ones.

This evidence leaves plenty of room for CPs to rationalize their advice on the most appropriate pharmacological treatments based on the LBP symptoms. Indeed, topical analgesics have been effective in the management of acute musculoskeletal pain, with an incidence of systemic or local adverse events equal to control [27]. Considering their efficacy/safety balance, topical analgesics could be used more frequently for acute LBP events with low/medium pain intensity as stand-alone or combination treatment. On the contrary, oral analgesics (e.g., paracetamol, opioids) and other medicines could be suggested by CPs (i.e., if OTC medicines are available on the market) or prescribed by the physicians for more intense acute and chronic LBP based on the existing guidelines [28].

Moreover, the combination of RMDQ and SBST improved the assessment process, allowing the CPs to properly identify patients with a high risk of persistent disability requiring a medical consult before a worsening of a patient’s condition. Patients with a low degree and risk of disability might be taken in care by the CPs and monitored. On the contrary, CPs should suggest consultation of physiotherapists and physicians in the presence of medium risk patients or send immediately the patients with high-risk.

In this context, it is noteworthy that the application of validated tools allowed the CPs to intercept patients who had never reported their problem to their physicians, despite having a high degree or high risk of disability. Thus, validated tools can be valuable in reducing the long-term expenditures of the healthcare system. This aspect is particularly relevant considering that 36.2% of patients have not reported their condition to the physicians. Among them, 4.1% and 5.1% had a high degree and risk of disability, respectively. The percentage reaches 31.4% selecting only patients that had never consulted either a physician or a physiotherapist (Table 1). Such findings confirmed the importance of CPs as essential “sentinel” healthcare professionals for a significant percentage of patients for which the community pharmacy seems to be the only point-of-care they visited to solve their LBP problem. 

A key-point of the role of community pharmacies in the management of LBP was the establishment of educational interventions to toughen the CPs’ knowledge and patients’ consciousness. In this field, several publications have stressed a good motivation and expertise of CPs on managing LBP [29,30]. However, the literature underlined some gaps in the CPs’ knowledge, especially on the relationship between LBP and physical exercises, that could be easily filled out by specific educational programs. Slater and colleagues demonstrated that the community pharmacy could be a feasible primary care portal in which evidence-based educational interventions can facilitate the return of pharmacy consumers to their daily life [12]. However, as demonstrated by Engers et al., the efficacy of educational interventions varies based on LBP types, intervention duration, and format [31]. For example, educational interventions of 2.5 h seemed to be particularly effective in patients with acute LBP, whereas they were not in those with chronic LBP. Patient educational intervention should always be part of a treatment program. Indeed, short patient education sessions or written information did not seem to be effective as a single treatment.

Although the investigation of patients’ beliefs was not one of the primary goals of the current study, the obtained data highlighted some interesting trends among the patients that could be useful for further educational intervention. Pharmacological interventions were more frequently used than non-pharmaceutical ones, although they were included in the guidelines for the management of both acute and chronic LBP as well [32]. Patients affected by chronic LBP seemed to have a more diversified approach in the treatment of their clinical conditions than those experiencing acute LBP, probably due to higher consciousness and/or knowledge of LBP. Indeed, the chronic LBP patients combined appropriate physical exercises and physiotherapist visits with the pharmacological treatments more frequently than other patients. Interestingly, the data showed that a higher propensity of females to rely on the physiotherapist consultation, which might be cultural-based or linked to higher satisfaction for the services as observed for inpatients [33].

**Figure 5:**
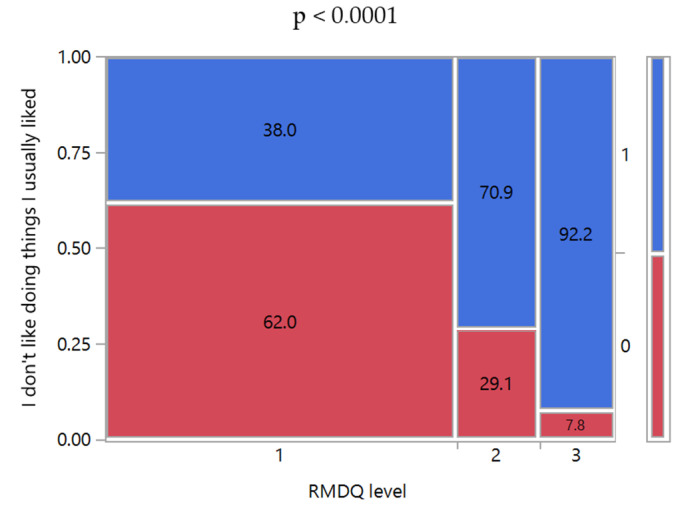
Mosaic plots of SBST Question No. 8 “In general, I don’t like doing things I usually liked” versus RMDQ level. SBST Question No. 8: 0, not agree; 1, agree. RMDQ levels: 1, low; 2, medium; 3, high. The class percentages were reported inside the mosaic plots. Above the graph is the *p*-value of Pearson’s chi-squared test between the two variables.

However, some limitations of the study are noteworthy. Although community pharmacists included in SGCP were selected to have a homogeneous and representative distribution throughout Italy, their number is limited. Despite the high number of patients enrolled in the study, the results cannot be considered representative of the entire Italian population of LBP patients, but they are limited to those visiting the community pharmacies in person to manage their LBP by pharmacological treatments (e.g., OTC medicines). Moreover, no information was collected about acute and chronic comorbidities other than those considered as exclusion criteria, although such data may be relevant for conducting a more comprehensive evaluation of the background of the patients’ attitudes to managing the LBP. The combination of RMDQ and SBST allowed a triage of LBP-affected patients but only provided tips about the impact of LBP on their quality of life. The influence of the pain/disability level on the quality of life of LBP patients has been reported in the literature [34,35]. However, a direct correlation has not always been evident, since clinical improvements might not be so significant to enhance the quality of life, or they could not be detectable due to biases [36,37]. In this context, the information could be derived from the study results. Indeed, the SBST questionnaire included questions focused on evaluating the patient’s attitudes to enjoy things they usually liked despite the LBP. As observed in Figure 5, the percentage of patients who enjoyed their favorite activities diminished significantly moving from low to high patient disability level, suggesting a reduction in their quality of life.

Another limitation of the study is the impossibility to investigate the causes of the significant reluctance of patients to visit their physician to report LBP. These aspects suggested the need for further studies focused on investigating which are the patients’ attitudes in interacting with healthcare professionals (e.g., physicians, pharmacists) for reporting health problems perceived by the patients as low or mild severity.

Despite such limitations, this study highlighted the fundamental role of community pharmacies as points of care, enabling healthcare systems to be able to meet and take care of patients daily who visit their community pharmacy. The involvement of CPs in non-dispensing services and pharmaceutical care programs has been quite common in the Anglo-Saxon world. Although further studies are needed for demonstrating the long-term effect of CPs interventions to support patients in LBP management and to improve their quality of life, this study confirmed the importance of exporting the same model of healthcare assistance in other systems and countries. 

## 5. Conclusions

The current observational study demonstrated the feasibility of the use of validated tools by Italian CPs to detect and triage patients affected by acute and chronic LBP. The collected data yielded a real-world cross-sectional view of patients with LBP seeking advice from CPs, underlining that the community pharmacy was the point-of-care to which about one-third of the patients (31.4%) turned to solve their problem. The administration of validated tools in community pharmacies allowed the CPs to assess the clinical condition of the patient, and to intercept undiagnosed patients. The obtained information may be useful for CPs to provide better advice and support. In the investigated population, most of the patients experienced a moderate intensity LBP (score range 4–7) and a low degree of disability (69%). About 15% of the patients were invited to consult a physician for the first time, although the degree of disability induced by LBP was medium or high. It confirmed the important sentinel role of CPs in intercepting patients that need medical support to manage their clinical condition and in redirecting them to the most appropriate healthcare professionals for a consultation (e.g., medical specialists in rheumatology, physicians, physiotherapists). Moreover, CPs have an important educational role in training patients on the proper use of self-medication treatment and management of LBP. The overall results unquestionably open new perspectives for the non-dispensing role of the Italian CPs as healthcare professionals who can collect epidemiological data, intercept patients with a red flag, and support physicians in the management and monitoring LBP patients.

## Figures and Tables

**Table 1:** Demographics of the study population at baseline and patients’ attitudes in low back pain (LBP) management [^a^ median (IQR); ^b^ mean ± St. Dev.].

Variable	Total	Females	Males	*p*-Value
No (%)	872	491 (56.3%)	381 (43.7%)	-
Age (years) ^a^	46 (36–56)	46 (35–55)	46 (38–57)	0.2775
Height (cm) ^b^	167 ± 9	163 ± 6	174 ± 7	<0.0001
Weight (kg) ^b^	71 ± 15	64 ± 13	80 ± 13	<0.0001
BMI (kg/m^2^) ^b^	25.0 ± 4.4	24.1 ± 4.5	26.3 ± 3.8	<0.0001
Smokers	212 (24.3%)	101 (20.6%)	111 (29.1%)	0.0036
Alcohol consumers	168 (19.3%)	50 (10.2%)	118 (31.0%)	<0.0001
LBP duration (days) ^a^	7 (3–15)	7 (3–15)	7 (3–15)	0.0382
LBP event within 6 months before interview	642 (73.6%)	372 (75.8%)	270 (70.9%)	0.1036
LBP reported to physician	556 (63.8%)	312 (64.5%)	244 (64.0%)	0.8793
Physiotherapist visit	285 (32.7%)	178 (36.3%)	107 (28.1%)	0.0107
Neither LBP reported to physician nor physiotherapist visit	274 (31.4%)	155 (31.6%)	119 (31.2%)	0.9443
LBP reported to physician and physiotherapist visit	243 (27.8%)	154 (31.4%)	89 (23.4%)	0.0024
Use of oral medicines	606 (69.5%)	353 (71.9%)	253 (66.4%)	0.0807
Use of topical medicines	501 (57.4%)	278 (31.9%)	223 (25.6%)	0.5713
Physical exercises	343 (39.3%)	194 (39.5%)	149 (39.1%)	0.9037

**Table 2:** The intensity of LBP based on a Pain Rating Scale (0: pain absence, 10 the worst pain possible), and patient distribution (No.; %) based on Roland and Morris Disability Questionnaire (RMDQ) and Start Back Screening Tool (SBST).

	Pain Rating Scale			
Pain Intensity	Total	Females	Males	*p*-Value
During the interview	6 (4–7)	6 (4–7)	5 (4–7)	0.6661
Within 24 h before (min)	3 (2–5)	3 (2–5)	3 (2–4)	0.2651
Within 24 h before (max)	7 (6–8)	7 (6–9)	7 (6–8)	0.1490
Disability	**Roland–Morris Disability Questionnaire**			
Low	602 (69.0%)	338 (68.8%)	264 (69.3%)	0.9543
Medium	141 (16.2%)	81 (16.5%)	60 (15.7%)	
High	129 (14.8%)	72 (14.7%)	57 (15.0%)	
Risk of disability	**Start Back Screening Tool**			
Low	411 (47.1%)	223 (45.4%)	188 (49.3%)	0.3388
Medium	314 (36.0%)	178 (36.3%)	136 (35.7%)	
High	147 (16.9%)	90 (18.3%)	57 (15.0%)	

## References

[1] Andersson G. (1998). Epidemiology of low back pain. Acta. Orthop. Scand. Suppl..

[2] James S.L., Abate D., Abate K.H., Abay S.M., Abbafati C., Abbasi N., Abbastabar H., Abd-Allah F., Abdela J., Abdelalim A. (2018). Global, regional, and national incidence, prevalence, and years lived with disability for 354 Diseases and Injuries for 195 countries and territories, 1990–2017: A systematic analysis for the Global Burden of Disease Study 2017. Lancet.

[3] Jarvik J.G., Hollingworth W., Heagerty P.J., Haynor D.R., Boyko E.J., Deyo R.A. (2005). Three-year incidence of low back pain in an initially asymptomatic cohort: Clinical and imaging risk factors. Spine.

[4] Walker B.F. (2000). The prevalence of low back pain: A systematic review of the literature from 1966 to 1998. J. Spinal. Disord..

[5] Burton A.K., Balagué F., Cardon G., Eriksen H.R., Hänninen O., Harvey E., Henrotin Y., Indahl A., Lahad A., Leclerc A. (2005). How to prevent low back pain. Best Pract. Res. Clin. Rheumatol..

[6] Grabovac I., Dorner T.E. (2019). Association between low back pain and various everyday performances: Activities of daily living, ability to work and sexual function. Wien. Klin. Wochenschr..

[7] van Tulder M.W., Koes B.W., Bouter L.M. (1995). A cost-of-illness study of back pain in The Netherlands. Pain.

[8] Hart L.G., Deyo R.A., Cherkin D.C. (1995). Physician office visits for low back pain: Frequency, clinical evaluation, and treatment patterns for a U.S. National Survey. Spine.

[9] Gyllenhammer D., Sickinger M., Gyllenhammer R.T. (2017). The Community Pharmacist’s Role in Managing Lower Back Pain. US Pharm..

[10] Dineen-Griffin S., Cert G., Garcia-Cardenas V., Rogers K., Williams K., Benrimoj S.I. (2019). Evaluation of a collaboratorive protocolized approach by community pharmacists and general medical practitioners for an Australian minor ailments scheme: Protocol for a cluster randomized controlled trial. JMIR Res. Protoc..

[11] Aly M., Garcia-Cardenas V., Williams K., Benrimoj S.I. (2018). A review of international pharmacy—Based minor ailment schemes a substitute for other service providers? A systematic review. Res. Soc. Adm. Pharm..

[12] Slater H., Briggs A.M., Watkins K., Chua J., Smith A.J. (2013). Translating evidence for low back pain management into a consumer—Focused resource for use in community pharmacies: A cluster-randomized controlled trial. PLoS ONE.

[13] Miggos S., Giua Marassi C. (2017). Mal di schiena (Lombalgia). Inquadramento Clinico e Gestione dei Disturbi Minori in Farmacia.

[14] Donovan M.I. (1990). Pain: Clinical manual for nursing practice. J. Pain Symptom Manag..

[15] Thong I.S.K., Jensen M.P., Miró J., Tan G. (2018). The validity of pain intensity measures: What do the NRS, VAS, VRS, and FPS-R measure?. Scand. J. Pain.

[16] Hill J.C., Dunn K.M., Lewis M., Mullis R., Main C.J., Foster N.E., Hay E.M. (2008). A primary care back pain screening tool: Identifying patient subgroups for initial treatment. Arthritis Care Res..

[17] Roland M., Morris R. (1983). A Study of the Natural History of Back Pain. Spine.

[18] Roland M., Fairbank J. (2000). The Roland-Morris disability questionnaire and the Oswestry disability questionnaire. Spine.

[19] Maggiani A., Abenavoli A. (2019). Italian Translation and Cross-Cultural Adaptation of a Back Pain Screening Questionnaire (Start Back Screening Tool). Ann. Ig..

[20] Padua R., Padua L., Ceccarelli E., Romanini E., Zanoli G., Bondì R., Campi A. (2002). Italian version of the Roland Disability Questionnaire, specific for low back pain: Cross-cultural adaptation and validation. Eur. Spine J..

[21] Chapman J.R., Norvell D.C., Hermsmeyer J.T., Bransford R.J., DeVine J., McGirt M.J., Lee M.J. (2011). Evaluating common outcomes for measuring treatments success for chronic low back pain. Spine.

[22] National Heart, Lung and Blood Institute Assessing Your Weight and Health Risk. http://www.nhlbi.nih.gov/health/educational/lose_wt/risk.htm.

[23] National Institute of Neurological Disorder and Stroke Low Back Pain Fact Sheet. https://www.ninds.nih.gov/Disorders/Patient-Caregiver-Education/Fact-Sheets/Low-Back-Pain-Fact-Sheet.

[24] Manfrin A., Tinelli M., Thomas T., Krska J. (2017). A cluster randomized control trial to evaluate the effectiveness and cost-effectiveness of the Italian medicines use review (I-MUR) for asthma patients. BMC Health Serv. Res..

[25] Ernst M.E.E., Doucette W.R., Dedhiya S.D., Osterhaus M.C., Kumbera P.A., Osterhaus J.T., Townsend R.J. (2001). Use of point-of-service health status assessment by community pharmacists to identify and resolve drug-related problems in patients with musculoskeletal disorders. Pharmacotherapy.

[26] Silcock J., Moffett J.K., Edmondson H., Waddell G., Burton A.K. (2007). Do community pharmacists have the attitudes and knowledge to support evidence based self-management of low back pain?. BMC Muscoloskelet. Disord..

[27] Derry S., Wiffen P.J., Kalso E.A., Bell R.F., Aldington D., Phillips T., Gaskell H., Moore R.A. (2017). Topical analgesics for acute and chronic pain in adults—An overview of Cochrane Reviews. Cochrane Database Syst. Rev..

[28] Oliveira C.B., Maher C.G., Pinto R.Z., Traeger A.C., Lin C.-W.C., Chenot J.-F., van Tulder M., Koes B.W. (2018). Clinical practice guidelines for the management of non-specific low back pain in primary care: An updated overview. Eur. Spine J..

[29] Abdel Shaheed C., Maher C.G., Mak W., Williams K.A., McLachlan A.J. (2015). The effect of educational intervention on pharmacists’ knowledge, attitudes, and beliefs towards low back pain. Int. J. Clin. Pharm..

[30] Patel T., Chang F., Mohammed H.T., Raman-Wilms L., Jurcic J., Khan A., Sproule B. (2016). Knowledge, perceptions and attitudes toward chronic pain and its management: A cross-sectional survey of frontline pharmacists in Ontario, Canada. PLoS ONE.

[31] Engers A.J., Jellema P., Wensing M., van der Windt D.A.W.M., Grol R., van Tulder M.W. (2008). Individual patient education for low back pain. Cochrane Database Syst. Rev..

[32] Shipton E.A. (2018). Physical therapy approaches in the treatment of low back pain. Pain Ther..

[33] Vanti C., Pillastrini P., Monticone M., Ceron D., Bonetti F., Piccarreta R., Guccione A., Violante F.S. (2014). The Italian version of the Physical Therapy Patient Satisfaction Questionnaire—[PTPSQ-I(15)]: Psychometric properties in a sample of inpatients. BMC Musculoskelet. Disord..

[34] Mutubuki E.N., Beljon Y., Maas E.T., Huygen F.J.P.M., Ostelo R.W.J.G., van Tulder M.W., van Dongen J.M. (2020). The longitudinal relationships between pain severity and disability versus health-related quality of life and costs among chronic low back pain patients. Qual. Life Res..

[35] Guclu D.G., Guclu O., Ozaner A., Senormanci O., Konkan R. (2012). The relationship between disability, quality of life and fear-avoidance beliefs in patients with chronic low back pain. Turk. Neurosurg..

[36] Wettstein M., Eich W., Bieber C., Tesarz J. (2019). Pain Intensity, Disability, and Quality of Life in Patients with Chronic Low Back Pain: Does Age Matter?. Pain Med..

[37] Kovacs F.M., Abraira V., Zamora J., Teresa Gil del Real M., Llobera J., Fernández C., Bauza J.R., Bauza K., Coll J., Cuadri M. (2004). Kovacs-Atención Primaria Group. Correlation between pain, disability, and quality of life in patients with common low back pain. Spine.

